# The Other Face of Insulin—Overdose and Its Effects

**DOI:** 10.3390/toxics10030123

**Published:** 2022-03-03

**Authors:** Szymon Rzepczyk, Klaudia Dolińska-Kaczmarek, Aleksandra Uruska, Czesław Żaba

**Affiliations:** 1 Forensic Medicine Departament, Poznan University of Medical Sciences, 60-781 Poznan, Poland; szymon.rzepczyk@interia.eu (S.R.); czaba@ump.edu.pl (C.Ż.); 2 Department of Internal Medicine and Diabetology, Poznan University of Medical Sciences, 60-834 Poznan, Poland; kldiab@raszeja.poznan.pl

**Keywords:** homicide-suicide, insulin, diabetes mellitus, overdose, hypoglycemia

## Abstract

Insulin is the most effective glycemic-lowering drug, and for people suffering from type 1 diabetes it is a life-saving drug. Its self-dosing by patients may be associated with a higher risk of overdose, both accidental and deliberate. Insulin-induced hypoglycemia causes up to 100,000 emergency department calls per year. Cases of suicide attempts using insulin have been described in the literature since its introduction into therapy, and one of the important factors in their occurrence is the very fact of chronic disease. Up to 90% of patients who go to toxicology wards overdose insulin consciously. Patients with diabetes are burdened with a 2–3 times higher risk of developing depression compared to the general population. For this reason, it is necessary to develop an effective system for detecting a predisposition to overdose, including the assessment of the first symptoms of depression in patients with diabetes. A key role is played by a risk-conscious therapeutic team, as well as education. Further post-mortem testing is also needed for material collection and storage, as well as standardization of analytical methods and interpretation of results, which would allow for more effective detection and analysis of intentional overdose—both by the patient and for criminal purposes.

## 1. Introduction

Insulin is an anabolic hormone produced by pancreatic β-cells [1,2]. Its discovery in 1921 is considered one of the most outstanding medical achievements of the last century. In the following years, work on synthesizing this hormone continued, especially as it became clear in the 1970s that the demand was so great that extraction from bovine and porcine pancreases was insufficient [3]. Basal insulin secretion is the amount of insulin secreted in the fasting state in the absence of exogenous stimulation; it keeps the body’s metabolism in an anabolic state. Around 50% of the total insulin secreted by a healthy pancreas is secreted under basal conditions. After food intake, β-cells, driven by direct glucose sensing by glucose transporter type 2 (GLUT2) (as well as by neuronal signals and incretin signaling), release insulin into the blood to promote the absorption of carbohydrates, proteins, peptides, and lipids into other cells. The effect of insulin on peripheral glucose uptake in the muscles and rapid inhibition of gluconeogenesis and glycogenolysis in the liver results in a decrease of glucose levels in blood, which causes the β-cells to stop synthesizing and secreting insulin [2,4,5,6]. In addition to basal secretion, there is a surge of insulin release after a meal to prevent hyperglycemia.

## 2. Insulin Therapy

Insulin is the most effective drug in lowering glycemia, and for patients with type 1 diabetes (T2D), it is a life-saving treatment [7]. Therefore, it is important to understand its effects, clinical relevance, and the potential risks associated with taking it. Many types of human insulin and synthetic insulin analogs are now available for precise glycemic control. The ideal insulin therapy should mimic insulin secretion by a healthy pancreas as closely as possible [3,8,9]. Insulins can be divided into fast (e.g. Lispro, Aspart), short, intermediate, and long-acting (e.g. Degludec, Glargine) [10,11]. Fast-acting insulins replace per-meal injection. Long-acting insulins mimic basal insulin secretion [3]. Insulin therapy can take many forms depending on the patient’s needs and abilities. The simplest therapy is to use only basal insulin or only rapid-acting insulin (in the form of boluses). A common therapy is the so-called basal-bolus or the use of basal, long-acting insulin with rapid-acting insulin. In T1D, basal-bolus is virtually always the treatment of choice, preferably in an intensive insulin therapy model with bolus dose adjustment according to the specific meal, current blood glucose and planned physical activity. In the treatment of type 2 diabetes (T2D), if insulin therapy is required, a single injection of basal insulin is usually used initially, but full insulin therapy is often ultimately required [7]. For elderly people, who cannot cope with the method of multiple injections, it is possible to use mixed preparations. A characteristic feature of exogenous insulin used in the treatment of diabetes is its therapeutic index [12,13]. The use of insulin in a dose higher than the therapeutic dose may result in hypoglycemia. The onset, duration, and level of hypoglycemia depend on the dose and type of overdosed insulin [14]. It is postulated that the insulin dose has a greater influence on the duration of hypoglycemia than its type, but there is no clear position on this issue [13,14]. The course of hypoglycemia caused by insulin overdose also depends on other factors, such as body weight, amount of body fat, and liver function [14]. Studies show that the dose does not affect the outcome of the treatment of an overdose [15]. The exact lethal insulin dose has not been determined [16]. In extreme cases, insulin overdose causes severe hypoglycemia resulting in loss of consciousness, coma, severe and irreversible brain injuries, or death [13,17]. 

Most importantly, modern insulin therapy should be focused on physician referral, patient self-monitoring and preventing hypoglycemia with new devices, for example, insulin infusion pumps connected to a continuous glycemia monitor. New devices play a great role in supporting patients—as we mentioned in *Hypoglycemia*, even hybrid insulin pumps that deliver both insulin and glucagon have recently been introduced to therapy. All of the above are necessary to reach therapeutic success in diabetes therapy. 

### 2.1. Routes of Administration and Dosage

Diabetes therapy monitoring contains measurement of glycated hemoglobin (HbA1c) and monitoring the frequency of hypoglycemic episodes. These factors influence the choice of therapy and dosage. During therapy it is extremely important to realize that the goal of achieving a normal HbA1c level could even be dangerous for the patient, because mortality is directly related to the number of hypoglycemic episodes [10,18,19]. 

One of the recommended models of insulin therapy is to administer insulin before each meal (fast-acting) and before going to bed and/or in the morning (long-acting) [8,18]. Practically the only way to administer insulin is by injection. Insulin can be administered intravenously or subcutaneously. In recent years, personal insulin pumps have gained popularity, which, after proper setting, administer the appropriate doses of the basal infusion of the preparation themselves; however, boluses still have to be programmed manually. Pumps should not be used in uneducated patients with a low intellectual level and no contact with a specialist clinic [18]. For years, research has been underway on an alternative application of insulin. One of the options was Exubera (from Pfizer, another preparation of this type is Afrezza). It is a fast-acting inhaled insulin indicated in patients with T2D more than T1D. One blister contains 1 mg of the preparation and corresponds to 3 IU [3,20]. Another route of administration is intranasal preparation, but although it causes an immediate response, it is short-lived [3]. It is not possible to administer insulin orally, but research on this form of administration is still ongoing [21]. Insulin doses are selected individually for the patient. The dosage depends on body weight, physical exertion and tissue sensitivity to insulin [10,11]. Most often, the dosage is based on the patient’s experience, and the doctor only supports the therapy by helping to determine the base dose and determining the target of glycemic values. In T2D, the basis of treatment is lifestyle modification with metformin monotherapy. Insulin therapy in T2D should be adapted to the patient’s capabilities; it is also necessary to take care of the education of the closest family members. It is important to inform patients with T2D about possible side effects of insulin therapy, for example gaining weight. As in T1D therapy, insulin is administered by the patient alone [10].

### 2.2. Overdose 

Toxic and lethal doses of insulin are dependent on the patient’s baseline glycemia. The toxic dose introduces the patient to a state of severe hypoglycemia, the treatment of which is discussed below. The threshold for hypoglycemic symptoms depends on many factors and is individual to the patient. The daily blood glucose levels on which the patient works and the speed at which the blood glucose level is lowered are crucial. Severe hypoglycemia is said to be when treatment requires the help of another person [10]. For each patient, the threshold for the onset of severe hypoglycemia is different. The same applies to the lethal dose—there is no conclusive data on its value in the literature because it depends on many factors, such as baseline glycemia. Insulin-induced hypoglycemia is the cause of approximately 100,000 emergency department visits per year [22]. Severe hypoglycemia accounts for up to 10% of deaths among young people with type 1 diabetes [23].

### 2.3. Hypoglycemia 

Clinical hypoglycemia is a condition in which blood glucose levels fall below 70 mg/dL (3.9 mmol/L), and clinically important hypoglycemia is defined as glucose below 54 mg/dL (3 mmol/L). The result of glycemia should always be compared with the patient’s condition—symptoms of hypoglycemia may occur even with normal test results in a situation where glucose levels are lowered too quickly [18]. Hypoglycemia is more common in patients with T1D than with T2D. A study led by researchers at the University of Dundee found that among T1D patients receiving therapy, the incidence of hypoglycemia was almost 43 episodes per person per year. For comparison, in the studied group of T2D patients treated with insulin, the number of hypoglycemic episodes per year was 16 [24] (see Table 1 for more details). 

In T2D, people using oral medications such as sulfonylureas are more likely to be exposed to hypoglycemia [11,24]. Risk factors for hypoglycemia in patients with T2D include, to a large extent, dietary errors (e.g., skipping meals or too low carbohydrate supply), as well as physical exertion, stress, alcohol consumption, or drug abuse [25,26]. Interestingly, the risk of hypoglycemia does not increase in patients using both insulin and metformin [26,27]. In patients with T1D and T2D treated intensively, risk factors for hypoglycemia include intensive insulin therapy, better glycemic control [28], physical activity, dietary errors, and alcohol consumption. In addition, patients with a long history of T1D should be expected to have less awareness of hypoglycemia and more significant glycemic fluctuations, which are also risk factors for hypoglycemia [29]. In order to reduce the risk of hypoglycemia, it is necessary to educate the patient about risk factors, dosage, insulin supply, and glycemic monitoring. Equally important is the education of the patient’s family and his immediate environment (in the workplace or classroom). Hypoglycemia can be mild (correctable by the patient by, for example, taking food or a sugar-sweetened drink) or severe, requiring the help of another person. Symptoms of severe hypoglycemia are confusion, drowsiness, impaired coordination, visual disturbances, paresthesia, and finally, coma. If left untreated, it can lead to the death of the patient. Complications of hypoglycemia include brain damage, convulsions, stress cardiomyopathy syndrome, pulmonary edema, arrhythmias, such as bradycardia, and ventricular arrhythmias associated with QT prolongation [30]. Severe hypoglycemia should be treated with an infusion of IV 20% glucose at a dose of 0.2 g glucose/kg b.m., followed by an infusion of 10% glucose until consciousness is regained. In justified cases, 1 mg of SC or IM glucagon should be given and—in the absence of improvement— should be repeated after 10 min. In addition to glycemic compensation, other measures should be taken to stabilize the patient’s condition, depending on the co-occurring disorders (e.g., fluid rolling, electrolyte disturbance compensation, maintenance of diuresis) [10,27,31]. Furthermore, hybrid insulin pumps that deliver both insulin and glucagon have recently been introduced to support patients who are unaware of hypoglycemia. 

The signs and symptoms of insulin overdose are those of hypoglycemia caused by any factor [14,32]. A significant therapeutic difficulty after an overdose of exogenous insulin is its high molar mass, which prevents elimination using dialysis [15]. However, its proper treatment rarely results in death [33]. In addition, in times of less-advanced diagnostic tools, patients with hypoglycemia of unknown origin risked a prophylactic laparotomy to assess the occurrence of insulinoma [34]. Development of hypoglycemia is estimated to be from 20 min to 10 h after exogenous insulin intake [35].

It is important to remember that hypoglycemia increases risk of cardiovascular events [36]. This dependence has been observed in medical mega-trials performed on the general population of TD2 patients (ACCORD, VADT). An intensive antihyperglycemic therapy can also increase the risk of severe hypoglycemia. It is worth to notice the UKPDS trial, which did not demonstrate a significant reduction of macrovascular events during the intensive treatment. Furthermore, it showed that the benefits of an intensive strategy to control blood glucose levels appeared even 10 years after the end of treatments. However, the UKPDS study population was, with respect to ACCORD and VADT studies, younger, with less history of cardiovascular disease and neuropathy, lower baseline HbA1c, and lower risk of hypoglycemia [37]. 

Tight glycemic control (with insulin therapy) improves short- and medium-term cardiovascular outcomes in acute cardiovascular events in both diabetic and non-diabetic populations. What is also important is that tight glycemic control in acute cardiovascular diseases, such as acute coronary syndrome, exerts a cardioprotective action by, among others, anti-inflammatory and anti-apoptotic mechanisms, anti-oxidative stress, endothelium protection, FFA reduction, anti-glucotoxic effect, and heart stem cell protection [19]. Furthermore, stress-related hyperglycemia with cardial infraction is associated with an increased risk of in-hospital mortality in patients, both with or without diabetes. There is also increased risk of congestive heart failure or cardiogenic shock in patients with no diabetes. [19,34,35].

## 3. Accidents and Suicides 

The first description of a failed suicide attempt with exogenous insulin appears in literature in 1927, which is a few years after insulin was introduced into the treatment of diabetes in 1921 [16,38,39,40]. The first suicide committed with insulin was described in 1932 [40]. The overall suicides among diabetic people, even by purposefully overdosing insulin, are thoroughly analyzed and described in the scientific literature [32,41,42,43,44,45,46,47,48]. Any chronic disease is a factor, which increases the suicide rate for adults and children. Among the latter, the rate almost doubles [49,50]. Diabetes, as a chronic disease requiring self-control and long-term therapy, greatly increases the risk of suicide among patients [12,51,52]. The exact degree of increased suicide rate among diabetics is not precisely defined in published scientific works. However, it varies between different types of diabetes, and it is the worst in T1D [12,53,54,55]. Suicide by intentional insulin overdose is described as uncommon in medical publications [15]. Data indicate a significant underestimation of the number of suicide attempts and deaths from deliberate overdose [33,34,54,56]. Moreover, unsuccessful suicide attempts may sometimes be overlooked by the patient’s relatives and doctors [57]. From the perspective of forensic science, differentiating an accident from suicide is also often difficult and requires careful investigation [58,59,60]. Many people with diabetes experience hypoglycemia of unknown origin; some of these cases might be caused by a deliberate overdose of insulin in an attempt at suicide [12,61]. An analysis of insulin overdose cases collected in the toxicology department indicated that nearly 90% of them were suicidal overdoses, and only 5% accidental overdoses [12,62]. Descriptions of accidental insulin overdoses by diabetic people rarely appear in documents since they usually concern minor overdoses, which hardly ever require hospitalization. Such incidents may occur due to choosing the wrong dose for a particular meal or exercise, skipping a meal, vomiting, excessive alcohol consumption by the person taking insulin, or as a result of memory impairment, dementia, or decreased vision in the patient, especially in geriatric populations [63]. The differences in the risk of accidental insulin overdose depending on the therapy model or the preparation used are not precisely described in the literature. Difficulty distinguishing a deliberate overdose from an accidental one stems from the same predisposing factors: mental illness or alcohol abuse. Psychological aspects are of exceptional importance [12]. Compared to the general population, diabetic patients are two to three times more prone to develop depression [12,54,64,65,66]. The likelihood of depression during the entire lifetime of a diabetic person is almost 30% [12,67,68]. It is important to acknowledge that this relationship works both ways [12,54,69]. This is especially true of type 2 diabetes. To some degree, this can be explained by aspects related to lifestyle [69] and the influence of pharmacological treatment. Antidepressants and anxiolytics affect the hunger and satiety centers; therefore, they are a common cause of obesity [70]. Analyses show that depression increases the risk of developing type 2 diabetes by 37% to 60%, and up to 65% concerning overall diabetes [54,71,72,73]. Effective diabetes therapy is based mainly on the patient’s self-control, which depends on their goodwill and mental health. Its deliberate abandonment may be regarded as an act of a self-destructive nature [12,74,75]. It is estimated that more than half of the depression cases among people with diabetes go undiagnosed [12,76]. Among the people with diabetes, the risk of depression recurrence within 5 years is as high as 80% [76]. The incidence of suicidal thoughts is also higher than the rest of the population, which is closely related to the conscientiousness of the therapy [12,48]. One of the factors indicating an increased risk of suicidal behavior is a diagnosed mental illness [46,50]. The relationship between depression and an increased risk of suicide is proven and generally known [77,78]. Depression as a comorbid condition occurs in a significant proportion of suicide cases in the diabetic population, including those who choose insulin overdose as a way to take their own lives [41,79]. Studies show that approximately 23% of diabetic patients have suicidal thoughts, the risk of committing suicide is 2–4 times higher than in the healthy population, and the death rate from suicide stands at about 9% [54,80,81,82]. Suicide rates vary for each type of diabetes: from 0.1–18.9% for type 2 diabetes and 2.8–12% for type 1, depending on the subgroup [54,83,84]. While committing suicide, the type 2 diabetes patients are, on average, approximately 15 years older than the ones with type 1 [54]. There are also reports of other mental illnesses that coexist with diabetes, which often lead to suicides using an excessive amount of insulin [14,38]. An analysis of a series of cases conducted in Japan revealed a pre-incident mental illness (depression, alcohol addiction, borderline personality disorder, autism-related disorders, delusional disorders) in 64% of suicidal overdoses of insulin [14]. Studies in the pediatric diabetic population show that suicide risk factors in this group may change over time as the patient grows up [50]. Almost 30% of children claim they had suicidal thoughts in the first weeks after hearing the diagnosis, more than half of whom considered suicide immediately after hearing the diagnosis [75]. During the 12-year observation of the pediatric group, as much as 60–70% of patients had suicidal thoughts, and 6.4% made a suicide attempt, some of them more than once [75,85,86]. Over 60% of them did not have a specific method of committing suicide, while among children with a thoughtful plan to take their own life, the most common was drug overdose [75]. The most common suicide method among them was insulin overdose. The average age during the first suicide attempt was 16.7 years old. [75]. In the pediatric population, adherence to the therapeutic regimen is also of great importance. Failing to obey the treatment recommendations and thoughts of suicide are often a sign of struggling to cope with the stress related to the chronic disease [75]. In addition, diagnosing the disease in its acute stage, combined with a negative perception of the disease in the pediatric population, increases the likelihood of self-destructive thoughts and behaviors, which may be an attempt to draw attention to oneself [75]. Some scientific publications also include cases of insulin overdose suicide attempts by non-diabetic people; however, they are less common and often concern relatives of diabetics [14,15,39,57,87,88,89,90,91]. Special cases are suicide attempts with the use of insulin by medical professionals who know and understand the effects of insulin on the body and its pharmacokinetics, and who have easier access to drugs. [39,57,92,93,94,95]. The literature also describes attempts to perform an extended suicide with the use of insulin [96]. The choice of insulin as a means of taking one’s life often comes from its availability among the people suffering from diabetes [12,41,79]. Insulin overdose is the most common suicide method among type 1 diabetes patients; it is less frequently used by type 2 diabetics [54]. Cases of suicidal overdose with insulin usually involve subcutaneous injection [15,32,41]; however, there are also known cases of intravenous insulin administration [57], with the use of an insulin pump [79], and oral intake [97]. In some cases, insulin is not the only substance used for suicide. Combinations of insulin with alcohol, hypoglycemic drugs, tranquilizers, anxiolytics, analgesics, antidepressants and beta blockers have been described [41,54,92,94,96]. The predominant type of insulin used for suicidal purposes is undefined, with availability seeming to play the most important role [12]. An analysis of the effects of insulin overdose for suicidal purposes shows that 75–95% of cases end in full recovery, 1–3% in permanent neurological deficits, and 1–3% in death [62,98] [see Table 2]. There are scientific works attempting to explain the possible relationship between insulin and suicidal behavior at the biochemical level [59]. It has been proven that decreased levels of serotonin in the central nervous system are associated with an increased risk of self-destructive behaviors [59]. However, the influence of factors related to diabetes (insulin, glucose, free fatty acids, cholesterol, tryptophan) that may influence the concentration of serotonin in the central nervous system requires further research and analysis [59].

## 4. Discussion

### 4.1. Myth of the “Perfect Crime”—Murder with Use of Insulin

For years, insulin has been showcased in media and pop culture as a perfect, unsolvable crime weapon [16,35,40,99]. This was due to the lack of appropriate diagnostic methods for detecting exogenous insulin and the undeveloped methods of assessing blood glucose levels until the 1960s [15,16,33]. The first murder with insulin as a weapon was reported and described in 1957 [40,98,100]. Currently, there are many studies in the literature on homicides committed with the use of insulin [40,101,102]. The variety of crimes committed using exogenous insulin, including serial murder, carried out mainly by people with at least basic medical knowledge, is striking [40,99,103,104,105,106].

Moreover, the murders are sometimes disguised as suicide [105]. The database shows that 66% of murderers who use insulin worked in healthcare facilities, and 78% were related to the victim [16]. There are also reports of murders using an insulin pump to administer other exogenous substances in lethal doses [104]. Even though homicide with exogenous insulin is classified as rare, such cases are becoming increasingly frequent [99].

### 4.2. Other Aspects of Insulin Misuse

From the point of view of forensic science, it is essential to analyze the acts performed in a state of limited consciousness, which is a result of hypoglycemia caused by insulin abuse and overdose [107,108]. There are known cases of using the state of reduced awareness due to hypoglycemia as an argument of defense in cases involving violence or road accidents [107,108]. The literature describes cases of the use of insulin by athletes as an anabolic doping agent accelerating the increase of muscle mass, which can be used, for example, in bodybuilding [51,109,110,111,112]. In addition, there have been instances of insulin abuse for recreational purposes, to obtain pleasure from an induced hypoglycemic state [34,51]. Worth mentioning, also, are the cases of taking excessive doses of exogenous insulin in Munchausen syndrome [34,51,113]. In such situations, the victims may be people suffering from the syndrome by self-administrating insulin, or their closest relatives, typically children, in cases of Munchausen syndrome by proxy [51,114,115]. Therefore, it is important to educate and constantly re-educate patients and make sure that the therapeutic team responsible for the patient is vigilant, aware of the dangers, and able to detect, diagnose, and treat them.

### 4.3. Diagnostics of Exogenous Insulin Overdose

Postmortem peripheral blood glucose assessment may lead to false conclusions due to the physiological glucose drop of 1–2 mmol/L per hour [16,99]. What is more, blood taken from the central vessels or the heart also does not allow for the determination of blood glucose before death, due to the increase of glucose in high concentration caused by the enzymatic breakdown of glycogen in the liver after death and its transfer into large vessels [16]. The assessment of the concentration of glucose in the vitreous body can only be used to rule out hypoglycemia, never to confirm it; however, at present, it is postulated that the test is of low value [16,35].

The assessment of the concentration of C-peptide in the blood in people with suspected insulin overdose is of significant diagnostic value, especially among T2D patients and non-diabetic cases of overdose [16,116]. The concentration of C-peptide drops too low due to negative feedback after administering exogenous insulin [16,117]. However, its concentration increases due to hypoglycemia caused, e.g., by the presence of an insulin-producing tumor or overdose of insulin secretagogues, which provide important clues about the origin of hypoglycemia [16]. This knowledge enables the understanding and interpretation of the diagnostically significant ratio of insulin to C-peptide [16,118]. Although the breakdown of proinsulin results in 1 mole of C-peptide per mole of insulin, under normal conditions, this ratio is greater than 1 and shows an approximate mealtime variation of 3:1 to 10:1 due to the different half-lives of insulin (3–6 min) and peptide C (10-20-30 min) in the blood [16,99,119]. In the case of exogenous insulin-induced hypoglycemia, the ratio drops below 1 [16]. There are only two cases where the ratio drops below 1: an autoimmune disease with insulin receptor antibody formation and Rabson–Mendenhall syndrome. In both cases, with regular C-peptide metabolism, the insulin elimination is reduced [35,120,121,122]. Interpretation of the C-peptide to insulin ratio can be made only in the case of the assessment of insulin and C-peptide concentrations from the same blood sample and in close relation to the blood glucose concentration, assuming the correctness of the laboratory results and the physiological metabolism of these hormones in the body [35]. When interpreting the results, it is also worth remembering the reduced amounts of endogenous insulin and C-peptide in type 1 diabetes, which can cause the results to have a lower diagnostic value [123].

Assessment of insulin concentration can be made by direct or indirect immunological method or by mass spectrometry, which offers the possibility of differentiating insulin analogs [16,33,35,51,124,125]. Proper sampling and storage are essential in this case [16]. At least 10 mL of blood should be drawn for EDTA or heparin as soon as possible before starting the treatment [35]. Then, the plasma should be separated; the time between blood collection and separation affects the quality of the sample [16]. Insulin and C-peptide are only stable in the collected sample at a minimum temperature of −20 °C; at room temperature, they are quickly destroyed [16,126]. It is also important to properly collect and store samples taken from each patient suspected of an overdose of insulin to secure any evidence for further analysis and investigation [105]. The assessment of proinsulin concentration in the protocol, as for the assessment of insulin and C-peptide concentrations, carries a diagnostic value [127]. Due to negative feedback, its concentration decreases after administering an exogenous insulin analog [16]. The exact amount of excess insulin and the timing of administration cannot be determined solely based on an estimate of hormone level [16]. The described methods of assessing hormone levels are only valid for blood samples collected from a live patient [16]. The tests of hormone concentrations from blood collected post-mortem should be treated appropriately and with caution due to the destructive effect of hemolysis and contamination with insulin and C-peptide due to their post-mortem release from the pancreas [16,33,128]. Damage to insulin disulfide bridges in the presence of hemoglobin is postulated [99,128]. Blood should be drawn from a peripheral vessel to avoid contamination with hormones released from the pancreas, and then analyzed in a protocol identical to that of a living person. The test can also be replaced with the determination of insulin in the vitreous body, however this method requires further research [51,117,129,130].

Attempts to assess the presence and concentration of insulin in the urine are not a viable diagnostic method [16]. Although insulin is filtered through the glomerulus, it is almost completely reabsorbed in the renal tubules, so the existence of insulin in the urine in people with abnormal reabsorption due to kidney damage could be misinterpreted as an insulin overdose. However, it is possible to analyze urine using mass spectrometry to determine the presence of breakdown products of synthetic insulin analogs in people in whom they should be absent [4]. In this case, too, urine should be stored at −20 °C [35].

When examining the corpses of people with reasonable suspicion of death due to hypoglycemia due to insulin overdose, it is important to look for the injection place [15,16]. After identifying a potential puncture spot, it is important to collect a sample, store it at −20 °C, and perform an immunohistochemical analysis for the presence of exogenous insulin [16,131]. At the injection spot, insulin can be detected many days after the event [95,128]. Insulin can build up in peripheral nerves, gaps between adipocytes, inflammatory cells, and connective tissue [99]. An autopsy usually does not reveal any specific macroscopic changes [33,51,57,105]. The histopathological examination of the samples taken from the brain shows a more significant proliferation of astrocytes [99]. It is also important to carefully assess the scene, mainly searching for needles, syringes, applicators, and insulin packs [4]. The analysis of the circumstances may provide the information necessary to differentiate between homicide, suicide, or an accident [51,99].

It is also essential to differentiate insulin overdose from sepsis or infections of various origins, especially those without diabetes [35]. In the case of sepsis, hypoglycemia occurs, and insulin and C-peptide concentrations are also low. However, in the presence of Escherichia coli infection, it may result in a misleadingly high insulin concentration measured by an immunoassay because of cross-reactions [16,40]. When interpreting the results of the immunological assessment of hormone levels, one should also bear in mind the possibility of the results getting altered due to cross-reactions with other substances present in the body and the different specificity of the immunoassay kits compared to the various insulin analogs currently used [35,132,133].

## 5. Conclusions

It is essential to develop an effective system that can quickly detect a predisposition to misuse of insulin. The most important aspect seems to be assessing the first symptoms of depression in diabetic patients to prevent the development of the disease and possible suicidal actions. The awareness and vigilance of the interdisciplinary therapeutic team, which should include a clinical psychologist, also plays a key role. In this case, actions supporting the patient’s mental health are more than justifiable, e.g., enabling regular psychological consultations or a support group and creating a particular scale for doctors to detect early symptoms of the disease and start the treatment as soon as possible. It is essential to continuously educate patients about the treatment of diabetes, along with the potential dangers, and the importance of regular medical check-ups and adherence to the therapeutic regimen. Another essential aspect is to use insulin pumps that have been modified to prevent the administration of doses dangerous to the patient’s life, and inform relatives about a suspected overdose incident. In addition, the considerable development of glucose monitoring systems gives hope in preventing side effects of the treatment of diabetes. Further research is needed to standardize the detection of post-mortem insulin overdose regarding the principles of specimen collection and storage, the analytical methods used, and the interpretation of the result. Defining straightforward algorithms for suspected insulin overdose in terms of therapeutic pathways and diagnostic methods is the key to saving patients’ lives and bringing justice by providing the basis for the effective detection of criminal insulin administration, as well as actions taken in a state of limited awareness as a result of hypoglycemia.

## Figures and Tables

**Table 1:** Frequency of hypoglycemia episodes based on University of Dundee researches [22].

Hypoglycemia Episodes	T1D	T2D
All hypoglycemia episodes per person per year	42.89	16.36
Severe hypoglycemic episodes per person per year	1.15	0.35
All recorded hypoglycemic events	336	236

**Table 2:** Insulin suicidal overdose effects.

Insulin Suicidal Overdose Effect	Full Recovery	Permanent Neurological Deficits	Death
Percentage [%]	75–95	1–3	1–3

## Data Availability

Not applicable.

## Copyrights

This is an original paper. Tables are original. No copyrights permissions included.
